# PBP1A Directly Interacts with the Divisome Complex to Promote Septal Peptidoglycan Synthesis in Acinetobacter baumannii

**DOI:** 10.1128/jb.00239-22

**Published:** 2022-11-01

**Authors:** Katie N. Kang, Joseph M. Boll

**Affiliations:** a Department of Biology, University of Texas Arlington, Arlington, Texas, USA; b Department of Immunology, University of Texas Southwestern Medical Center, Dallas, Texas, USA; Geisel School of Medicine at Dartmouth

**Keywords:** peptidoglycan, penicillin-binding protein, division, septation, elongation, Gram-negative, Gram-negative bacteria

## Abstract

The class A penicillin-binding proteins (aPBPs), PBP1A and PBP1B, are major peptidoglycan synthases that synthesize more than half of the peptidoglycan per generation in Escherichia coli. Whereas aPBPs have distinct roles in peptidoglycan biosynthesis during growth (i.e., elongation and division), they are semiredundant; disruption of either is rescued by the other to maintain envelope homeostasis and promote proper growth. Acinetobacter baumannii is a nosocomial pathogen that has a high propensity to overcome antimicrobial treatment. A. baumannii contains both PBP1A and PBP1B (encoded by *mrcA* and *mrcB*, respectively), but only *mrcA* deletion decreased fitness and contributed to colistin resistance through inactivation of lipooligosaccharide biosynthesis, indicating that PBP1B was not functionally redundant with the PBP1A activity. While previous studies suggested a distinct role for PBP1A in division, it was unknown whether its role in septal peptidoglycan biosynthesis was direct. Here, we show that A. baumannii PBP1A has a direct role in division through interactions with divisome components. PBP1A localizes to septal sites during growth, where it interacts with the transpeptidase PBP3, an essential division component that regulates daughter cell formation. PBP3 overexpression was sufficient to rescue the division defect in Δ*mrcA* A. baumannii; however, PBP1A overexpression was not sufficient to rescue the septal defect when PBP3 was inhibited, suggesting that their activity is not redundant. Overexpression of a major dd-carboxypeptidase, PBP5, also restored the canonical A. baumannii coccobacilli morphology in Δ*mrcA* cells. Together, these data support a direct role for PBP1A in A. baumannii division and highlights its role as a septal peptidoglycan synthase.

**IMPORTANCE** Peptidoglycan biosynthesis is a validated target of β-lactam antibiotics, and it is critical that we understand essential processes in multidrug-resistant pathogens such as Acinetobacter baumannii. While model systems such as Escherichia coli have shown that PBP1A is associated with side wall peptidoglycan synthesis, we show herein that A. baumannii PBP1A directly interacts with the divisome component PBP3 to promote division, suggesting a unique role for the enzyme in this highly drug-resistant nosocomial pathogen. A. baumannii demonstrated unanticipated resistance and tolerance to envelope-targeting antibiotics, which may be driven by rewired peptidoglycan machinery and may underlie therapeutic failure during antibiotic treatment.

## INTRODUCTION

The Gram-negative cell envelope consists of inner and outer membrane lipid bilayers, separated by a periplasmic space enriched with peptidoglycan (PG). The peptidoglycan sacculus determines the bacterial cell shape. Peptidoglycan biosynthesis and assembly are coordinated by the elongasome and divisome, two multiprotein complexes that regulate rod length and daughter cell formation, respectively. Dogma suggests that elongation and division activities are dependent on the lipid II substrate availability (1–4); therefore, rod shape and septum assembly are dictated by substrate competition between the two peptidoglycan synthase complexes. Increased elongasome activity favors narrow, elongated rods (5) with V-shaped division constrictions (4), whereas increased divisome activity results in wide, short cells (5) with blunted septal sites (4).

In proteobacteria like Escherichia coli, the divisome and elongasome complexes include more than 20 proteins that tightly regulate peptidoglycan biogenesis (6, 7). They include several regulatory components, the *s*hape, *e*longation, *d*ivision, and *s*porulation (SEDS) family glycosyltransferases FtsW and RodA and the class B penicillin-binding protein transpeptidases PBP2 and PBP3, which synthesize peptidoglycan along the cell axis and septum, respectively (8–12). The class *A p*enicillin-*b*inding *p*roteins (aPBPs) PBP1A and PBP1B (encoded by *mrcA* and *mrcB*, respectively) are primary peptidoglycan synthases that also contribute to side wall and septal peptidoglycan biosynthesis. aPBPs are bifunctional, with distinct domains that catalyze either transpeptidase or glycosyltransferase activities. In E. coli, PBP1A and PBP1B are functionally semiredundant. Individual *mrcA* and *mrcB* deletions do not contribute to measurable morphological defects (13, 14), and only one is required for viability (15). Importantly, only one aPBP is required for elongation (10) and division (16) in the well-studied model organism.

aPBPs directly interact with specific monofunctional transpeptidases in the elongasome or divisome. In E. coli, PBP1A associates with PBP2 in the elongasome (14). PBP2 also interacts with the monofunctional elongation glycosyltransferase RodA (17). Elongasome activity is regulated by MreBCD through interactions with the PBP2 cytoplasmic domain (17). In contrast, PBP1B associates with divisome transpeptidase, PBP3 (18, 19), and with FtsN (18), an essential bitopic membrane protein necessary to promote division (20) by inducing PBP3 activity (21). PBP1B forms a trimeric complex with PBP3 and FtsW, the monofunctional divisome glycosyltransferase homolog of RodA (19). FtsW inhibits PBP1B-mediated peptidoglycan polymerization in the absence of PBP3 (19). While PBP1A and PBP1B interact with distinct peptidoglycan assembly complexes, either aPBP can compensate for the other to restore the missing activity and function (14).

Our understanding of peptidoglycan synthases in Gram-negative bacteria are largely based on studies in the rod-shaped model organism, E. coli. However, accumulating evidence clearly shows that PBP1B activity is not functionally redundant with PBP1A in the highly drug-resistant nosocomial pathogen A. baumannii (22–24). Deletion of *mrcA*, which encodes PBP1A, caused septation defects, which induced cell chaining and cell filamentation. Moreover, PBP1A catalytic activity was necessary for completing septation (23). Consistent with a role in division, PBP1A was enriched at the midcell during growth, where divisome components assemble to regulate cell envelope invagination during cytokinesis. While phenotypes were consistent with a role for PBP1A in A. baumannii division, it was not determined whether PBP1A directly or indirectly interacted with the divisome machinery. Here, we show that A. baumannii PBP1B is not functionally redundant with PBP1A. Instead, PBP1A showed distinct localization at the midcell prior to division, where it presumably promotes septal peptidoglycan synthase activity. PBP1A complexes with PBP3 during growth, indicating that it directly contributes to divisome activity and daughter cell formation in A. baumannii. In contrast, we were unable to confirm direct interaction between PBP1A and PBP2 during growth. Further supporting an overlapping role between PBP1A and PBP3 enzymatic activity in A. baumannii PBP3 overexpression rescued the Δ*mrcA* division defect. In contrast, PBP1A could not rescue the septal defect when PBP3 was inhibited with aztreonam, suggesting that PBP1A and PBP3 have distinct roles in septal peptidoglycan biosynthesis. Together, these studies uncover a unique role for PBP1A in A. baumannii cell division and fitness.

## RESULTS

### PBP1B is not functionally redundant with PBP1A in A. baumannii.

**FIG 1:**
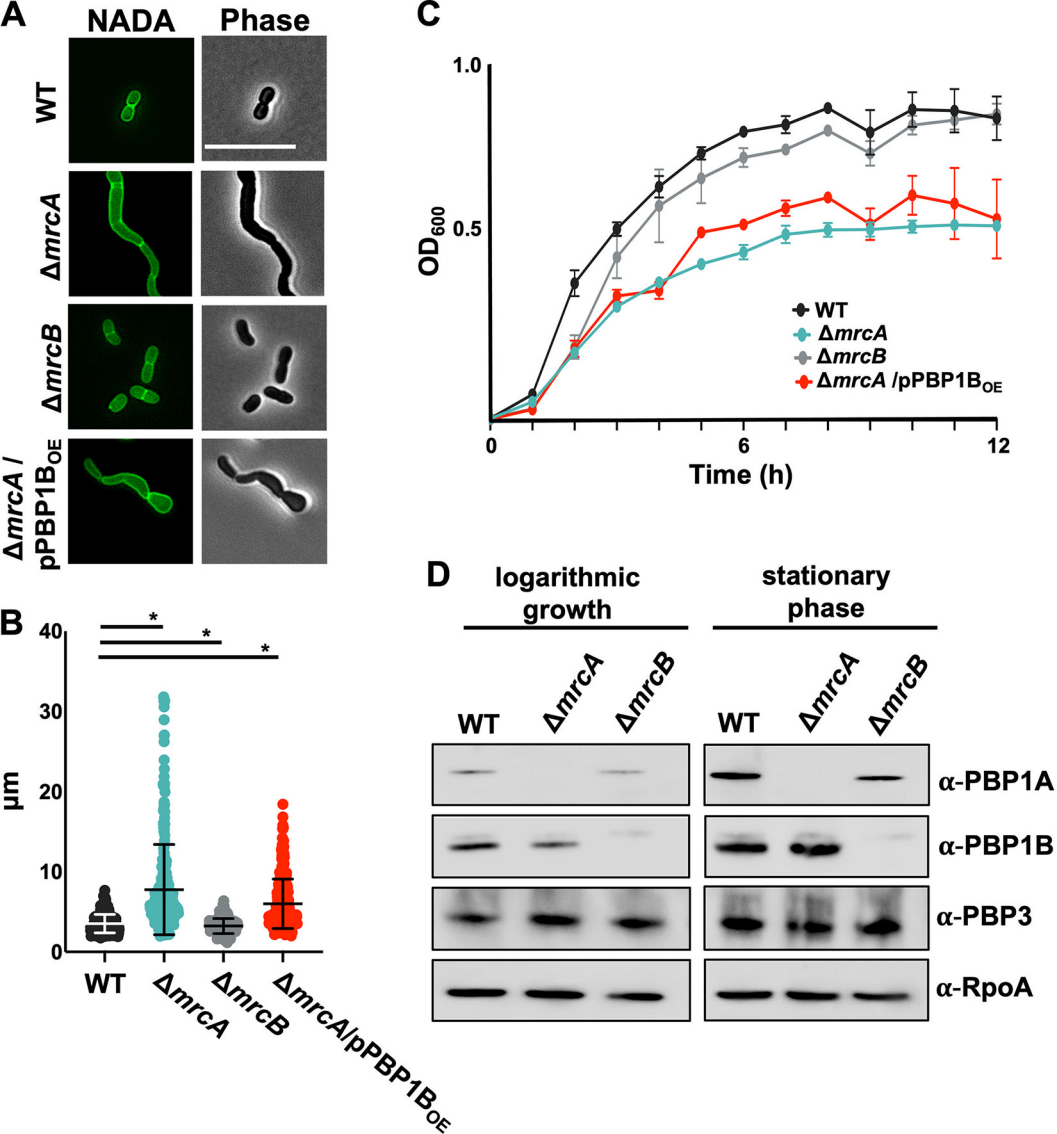
PBP1B cannot compensate for the division defect in Δ*mrcA* A. baumannii. (A) Fluorescence (left) and phase (right) microscopy of wild-type (WT), Δ*mrcA*, Δ*mrcB*, and Δ*mrcA*/PBP1B_OE_ cells. Scale bar = 10 μm. (B) Length (pole to pole) quantifications of each cell population (*n *≥ 300) were calculated using ImageJ software with the MicrobeJ plugin. Each dot represents one cell. The error bars represent the standard deviation. Significance testing was conducted using Student’s *t* test with two-tailed distribution assuming equal variance. *, *P < *0.05. (C) Optical density growth curve of WT, Δ*mrcA*, Δ*mrcB*, and Δ*mrcA*/PBP1B_OE_ cells. The error bars represent the standard deviation. OD_600_, optical density at 600 nm. (D) Western blot of WT, Δ*mrcA*, and Δ*mrcB* whole-cell lysates collected in mid-logarithmic growth (left) and stationary phase (right). PBP1A is 94.74 kDa; PBP1B is 88.21 kDa; PBP3 is 67.66 kDa; RpoA is 37.62 kDa.

In contrast to the proposed aPBP peptidoglycan synthase model in rod-shaped E. coli, previous data suggested that PBP1A has a primary role in division in coccobacilli-shaped A. baumannii cells and that PBP1B does not compensate when it is inactive (23). *mrcA* mutation induced an A. baumannii growth defect, cell chaining, and reduced fitness, whereas the Δ*mrcB* mutation did not. Consistent with these data, Δ*mrcA* A. baumannii expressing a transpeptidase-defective PBP1A_S459A_ (but not Δ*mrcB* or Δ*mrcB* expressing PBP1B_S455A_) impaired growth (24). To determine if A. baumannii PBP1B could rescue the Δ*mrcA* septation defect, we visualized cells using phase and fluorescence microscopy (Fig. 1A). As previously done (23, 25–27), cells were grown to the logarithmic growth phase and stained with the fluorescent d-alanine derivative NADA [NBD-(linezolid-7-nitrobenz-2-oxa-1,3-diazol-4-yl)-amino-d-alanine] to visualize the peptidoglycan. NADA is incorporated into peptidoglycan by PBPs and ld-transpeptidases (28–31). Consistent with previous reports (23), the Δ*mrcA* cells produced multiseptated chained cells relative to the wild type, while the Δ*mrcB* cells did not. Using an IPTG (isopropylthio-β-galactoside)-inducible PBP1B construct (see Fig. S1A in the supplemental material), denoted as pPBP1B_OE_, overexpression was not sufficient to rescue the septation defect in the Δ*mrcA* cells (Fig. 1A). Length quantifications also showed that PBP1B overexpression could not restore the septation defect in Δ*mrcA* A. baumannii (Fig. 1B).

To determine whether PBP1B expression could rescue the fitness defect in Δ*mrcA* cells, the growth rates of aPBP mutants were measured (Fig. 1C). Δ*mrcB* cells did not show significant growth defects relative to the wild type, and PBP1B expression was unable to rescue the growth defect in Δ*mrcA* A. baumannii.

Lastly, if PBP1B could compensate for the loss of PBP1A, we hypothesized that increased PBP1B levels might be evident in Δ*mrcA* cells. Using specific antisera, the PBP1A and PBP1B levels were compared in wild-type, Δ*mrcA*, and Δ*mrcB* cultures in logarithmic growth or stationary phase (Fig. 1D) and quantified (Fig. S1B). There was an obvious difference in aPBP levels between growth phases, where PBP1A and PBP1B expression increased in the stationary phase relative to the growth phase. Notably, the PBP1A levels in the Δ*mrcB* cells were lower than those in the wild type. Conversely, the PBP1B levels were decreased in the Δ*mrcA* cells. The changes in protein levels were reproducible and might illustrate a mechanism for maintaining proper stoichiometry between the two proteins, particularly if their properties are antagonistic. While it is possible that aPBP enzyme activity could increase when the other is defective, these data support a model where the PBP1B activity is not functionally redundant with PBP1A; specifically, PBP1B cannot restore Δ*mrcA* septation and fitness defects.

### Increased PBP1A activity shifts peptidoglycan biosynthesis toward division in A. baumannii.

**FIG 2:**
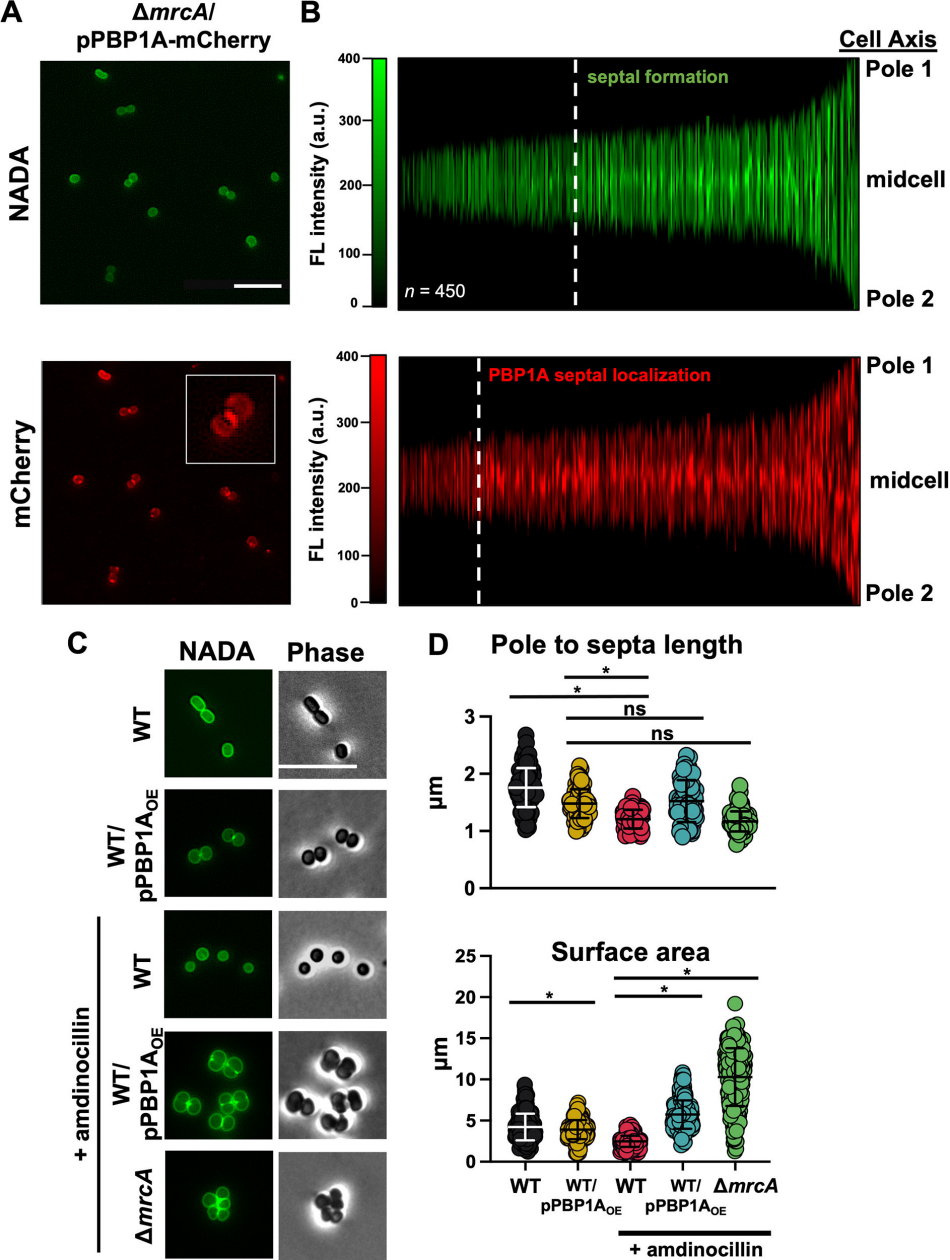
PBP1A shifts the peptidoglycan synthesis balance toward division. (A) Fluorescence microscopy of Δ*mrcA/*pPBP1A-mCherry cells with NADA fluorescence (top) and PBP1A-mCherry (bottom). PBP1A-mCherry is expressed by its native promoter. Scale bar = 10 μm. The inset (bottom) shows a representative cell at higher magnification. (B) Demographs depicting NADA fluorescence (top) and PBP1A-mCherry (bottom) intensity localization along the cell axis. Cells are ordered by increasing length (*n *= 450). Dashed lines indicate when midcell localization of NADA and PBP1A-mCherry becomes apparent. (C) Fluorescence and phase microscopy of wild-type (WT) and WT/PBP1A_OE_ (overexpression; inducible promoter) cells and WT, WT/PBP1A_OE_, and Δ*mrcA* cells treated with 0.5-MIC amdinocillin. Scale bar = 10 μm. (D) Length (pole to septa in dividing cells or pole to pole in nondividing cells) (*n *= 100) was calculated using ImageJ software. Area was calculated as surface square pixels using MicrobeJ. Each dot represents one cell. The error bars indicate the standard deviation. Significance testing was conducted using Student’s *t* test with two-tailed distribution assuming equal variance. *, *P < *0.05; ns, not significant.

Using a previously described (23) pPBP1A-mCherry reporter fusion, expressed from its native promoter (Fig. S2A), that fully complemented the division defect in Δ*mrcA* A. baumannii, we characterized PBP1A localization. While fusion constructs can include some caveats, expression showed diffuse PBP1A localization throughout the cell (Fig. 2A). Notably, fluorescence intensity was enriched at the midcell, where divisome proteins assemble to regulate septal formation and division. PBP1A-mCherry localization relative to septal peptidoglycan formation was compared using demographs (Fig. 2B). PBP1A-mCherry accumulated at the midcell prior to septal peptidoglycan assembly, suggesting that PBP1A not only contributes to septal peptidoglycan biogenesis but could also shift peptidoglycan synthesis from elongation to division.

In line with previous work (23), these data also show that PBP1A overexpression (via pPBP1A_OE_) induced cell rounding, a phenotype consistent with an increased septation rate and/or decreased elongation (Fig. 2C). Notably, PBP2 inhibition also produced rounded cells in *Enterobacterales* strains (32). Based on these data, we hypothesized that PBP1A activity promotes septation and/or antagonizes elongasome activity in A. baumannii.

PBP2 is specifically inhibited by the β-lactam amdinocillin (32), so A. baumannii cells treated with sub-MICs of amdinocillin expressing either native levels or overexpressing PBP1A (Fig. S2B), which also restored growth (Fig. S2C), were analyzed using fluorescence and phase microscopy (Fig. 2C). The distance from pole to septa (or pole to pole in nondividing cells) was measured among populations (*n *= 100) to quantitate it (Fig. 2D). Intriguingly, wild-type amdinocillin-dependent PBP2 inhibition produced statistically significant shorter cells relative to wild-type cells overexpressing PBP1A (Fig. 2D, top). While this could be a dosage effect, where PBP2 inhibits the elongasome more potently than PBP1A, it was not clear if PBP1A directly inhibited elongasome activity. Notably, cells treated with amdinocillin and overexpressing PBP1A were larger relative to amdinocillin-treated wild-type cells and cells overexpressing PBP1A (Fig. 2D, bottom). While an increase in cell surface area could suggest a synthetic effect from PBP1A overexpression in combination with amdinocillin treatment, an increased rate of septal peptidoglycan polymerization could also account for the increase in cell size, where cells are rapidly dividing before the prior division cycle is complete. To distinguish whether cells overexpressing PBP1A produced more cells overall, we calculated the CFU per milliliter over time (Fig. S2D). Cells overexpressing PBP1A reproducibly showed high CFU per milliliter relative to the wild type regardless of amdinocillin treatment, suggesting that increased septation may drive the formation of short round cells. In line with these data, Δ*mrcA* cells treated with a sub-MIC of amdinocillin were round and clumped (Fig. 2C), supporting a model where defects in both septation and elongation reduced the CFU relative to that of the wild type (Fig. S2D). Together, these data suggest that PBP2 inhibition was not sufficient to shift peptidoglycan biosynthesis toward division without PBP1A-dependent septal peptidoglycan activity.

### PBP1A directly interacts with PBP3 at the divisome during growth.

**FIG 3:**
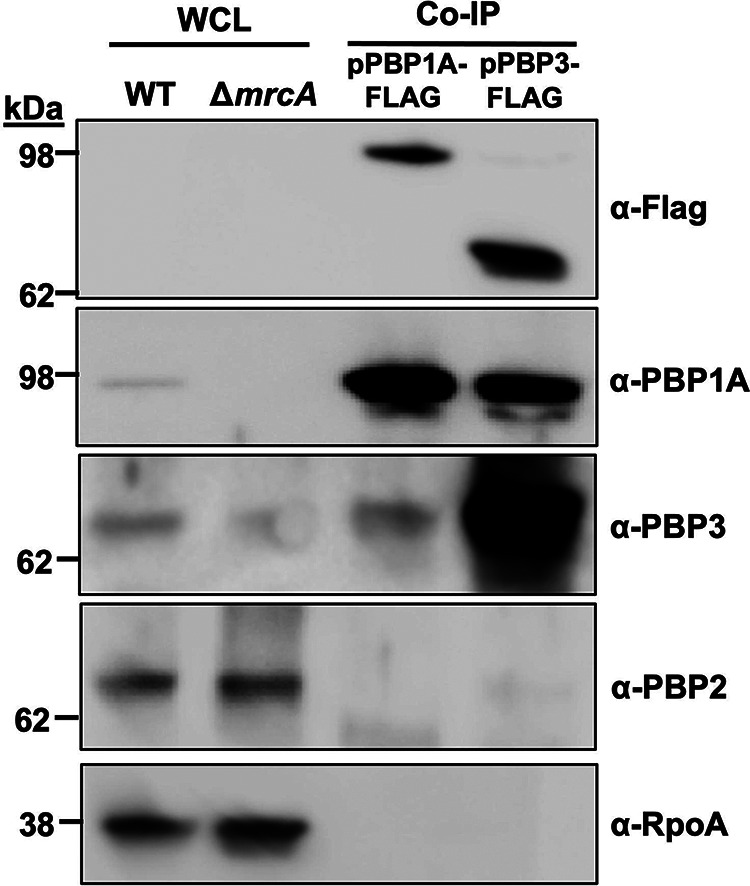
Detection of *in vivo* interactions between PBP1A and PBP3, but not PBP2. Immunoblots with whole-cell lysates (WCL) of wild type (WT) and Δ*mrcA* (left two lanes) were run as controls. The right two lanes contain analysis of PBP1A-FLAG and PBP3-FLAG coimmunoprecipitated (CoIP) A. baumannii proteins. A. baumannii cells were cross-linked with DSP [dithiobis(succinimidyl propionate)] to trap complexes and then quenched to stop the cross-linking reaction. Cells were pelleted, osmotically lysed, and then solubilized with a solution containing Triton X-100. After centrifugation, either PBP1A-FLAG or PBP3-FLAG was immunoprecipitated overnight with anti-FLAG M2 affinity gel resin. FLAG-tagged proteins were detected using a monoclonal anti-FLAG antibody. PBP1A, PBP3, and PBP2 were detected with specific antisera after immunoprecipitation. The first two lanes contain proteins from whole-cell lysates from wild-type (WT) or Δ*mrcA* A. baumannii. Antisera specific for RpoA was used as a control to show that cytoplasmic contamination was not present in the CoIP fractions. PBP1A is 94.74 kDa; PBP2 is 74.45 kDa; PBP3 is 67.66 kDa; RpoA is 37.62 kDa.

Previous work (23) and the PBP1A localization studies here are consistent with the accumulation of unproductive septal events in Δ*mrcA* cells, which implies that PBP1A promotes proper A. baumannii division. However, it was unclear whether PBP1A directly interacted with divisome proteins to promote septal peptidoglycan biosynthesis. Next, we tested whether PBP1A directly interacted with PBP2 and PBP3 *in vivo* using coimmunoprecipitation (CoIP) (33). A C-terminal Flag fusion (PBP1A-FLAG) that fully complemented the Δ*mrcA* mutant phenotype was expressed (Fig. S3A) in wild-type cells. Cells were incubated with Lomant’s reagent (dithiobis succinimidylpropionate; DSP). DSP is a membrane-permeable cross-linker that reacts with primary amine groups and contains a 12.0-Å spacer arm. After DSP cross-linking, PBP1A immunoprecipitation showed direct interaction with PBP3, but not PBP2, in A. baumannii during growth (Fig. 3). Furthermore, immunoprecipitation of PBP3-FLAG expressed in wild-type cells (Fig. S3A) also showed direct interaction with PBP1A (Fig. 3). PBP3 is a highly conserved divisome protein, known to contribute to daughter cell formation during septation (34, 35). Direct interaction between PBP1A and PBP3 during growth supports a model where PBP1A directly promotes A. baumannii septation. Unlike in rod-shaped bacteria, where PBP1A interacts with PBP2 to promote elongasome-dependent side wall peptidoglycan biosynthesis, our data strongly suggest that PBP1A directly interacts with the divisome to promote septal peptidoglycan biosynthesis in A. baumannii.

### PBP3- and PBP5-dependent rescue septation defects in Δ*mrcA* A. baumannii.

**FIG 4:**
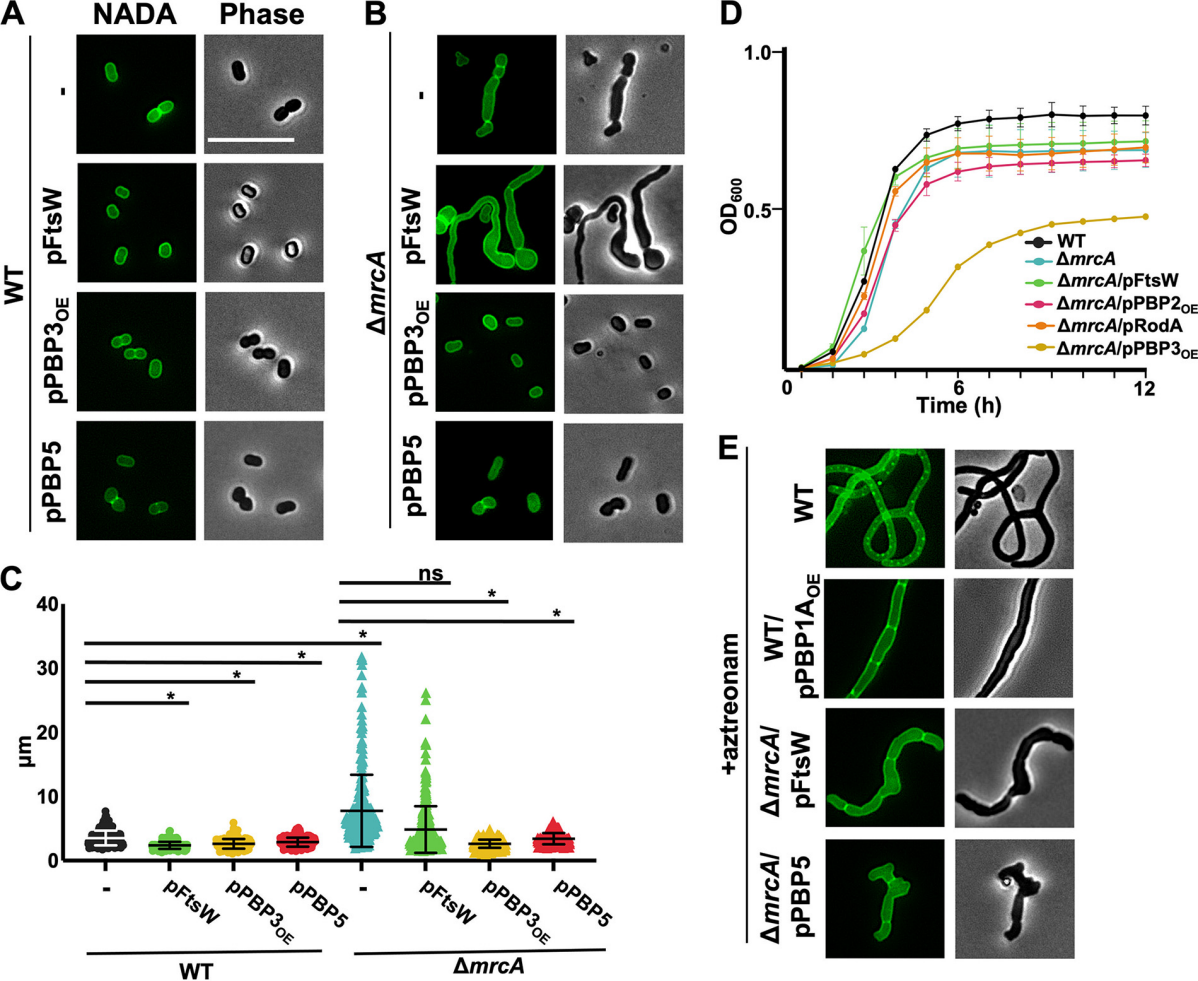
PBP3 and PBP5 rescue division in Δ*mrcA* A. baumannii. (A) Fluorescence and phase microscopy of wild type (WT), WT expressing FtsW, PBP3_OE_, and PBP5. Scale bar = 10 μm. (B) Fluorescence and phase microscopy of Δ*mrcA*, expressing FtsW, PBP3_OE_, and PBP5. (C) Length (pole to pole) (*n *≥ 300) was calculated using ImageJ software with the MicrobeJ plugin. Each dot represents one cell. Significance testing was conducted using Student’s *t* test with two-tailed distribution assuming equal variance. *, *P* < 0.05; ns, not significant. (D) Optical density (OD_600_) growth curve of wild type (WT), Δ*mrcA*, and Δ*mrcA* expressing various peptidoglycan synthases. The error bars indicate the standard deviation. (E) Fluorescence and phase microscopy of WT, WT expressing PBP1A_OE_, and Δ*pbp1A* expressing FtsW and PBP5, treated with sub-MIC aztreonam (8.0 mg/L).

Prompted by the direct interaction between PBP1A and PBP3, we hypothesized that PBP1A and PBP3, along with its glycosyltransferase partner, FtsW, worked together during septal peptidoglycan biogenesis. We overexpressed PBP3 (Fig. S3B) or FtsW in Δ*mrcA* cells to determine whether either divisome synthase (transpeptidase or glycosyltransferase) could restore the septation defect characteristic of Δ*mrcA* cells (Fig. 4). Fluorescence and phase microscopy showed that FtsW expression in wild-type cells (WT/pFtsW) produced shorter cells than the wild type (Fig. 4A), suggesting that the protein was expressed and induced an increased division rate. However, FtsW expression did not rescue the septal defects in Δ*mrcA* cells (Δ*mrcA*/pFtsW) and induced peptidoglycan bulging (Fig. 4B). In contrast, PBP3 overexpression (Δ*mrcA*/pPBP3_OE_) restored the canonical A. baumannii coccobacilli morphology (Fig. 4B). These data suggest that increased PBP3 activity could compensate for PBP1A defects to promote proper septal peptidoglycan biosynthesis, implying a similar function. Cell length quantifications (Fig. 4C) also supported our conclusion that PBP3 overexpression rescued septation defects in Δ*mrcA* cells, while FtsW expression did not.

Furthermore, several studies showed that PBP5, a dd-carboxypeptidase encoded by *dacA*, rescues division defects in PBP3-depleted E. coli (1, 36) and other bacteria (37). Considering that PBP3 rescued septation defects in Δ*mrcA* cells, we analyzed whether A. baumannii PBP5 could also rescue the septation defect in Δ*mrcA* cells. PBP5 expression (Δ*mrcA*/pPBP5) rescued the septation defect in Δ*mrcA* cells, like PBP3 overexpression (Fig. 4B). Cell length quantifications also supported our conclusion (Fig. 4C). While only transpeptidase (PBP3 and PBP5) expression rescued the division defect in the Δ*mrcA* strain, both FtsW and transpeptidase overexpression contributed to phenotypic changes in Δ*mrcA* A. baumannii cells. To determine whether these changes were specific to the loss of PBP1A activity, we overexpressed FtsW, PBP3, and PBP5 in wild-type cells (Fig. 4A). Expression of the divisome-associated PG synthases in the wild type produced shorter cells (Fig. 4A and C). However, in contrast to PBP1A overexpression, which promotes a spherical phenotype (Fig. 2C), the overexpression of divisome components maintained coccobacilli morphology (Fig. 4A). Together, these studies imply that in A. baumannii PBP1A, PBP3 and PBP5 could work together to coordinate proper septal peptidoglycan biogenesis.

### PBP3 and PBP1A do not share redundant septal peptidoglycan biosynthesis activity.

Previous studies from our lab (23) and others (24) demonstrated a significant fitness defect in Δ*mrcA* A. baumannii cells. We next asked whether restoring productive septation in Δ*mrcA* cells would be sufficient to rescue fitness. We overexpressed each of the monofunctional synthases in Δ*mrcA* cells and assessed their fitness using optical density growth curves (Fig. 4D). Intriguingly, PBP3 overexpression (Fig. S3B) was sufficient to rescue the septation defect in Δ*mrcA* cells (Fig. 4B and C) but not the fitness defect (Fig. 4D). However, overexpression of PBP3 in wild-type cells also produced a growth defect (Fig. S3C), suggesting that PBP3 overexpression is toxic regardless of PBP1A activity. Intriguingly, PBP2 expression (Fig. S3D) did not rescue the Δ*mrcA* fitness defect but was notably toxic when expressed in wild-type cells (expressing PBP1A).

We next asked whether PBP1A overexpression would similarly rescue division when PBP3 is disrupted. To disrupt PBP3, we treated cells with 0.5-MIC of aztreonam, which has high specificity for PBP3 and effectively prevents division in A. baumannii (23) and other Gram-negative bacteria (38), but we also cannot rule out off-target effects. When PBP1A was overexpressed in the aztreonam-treated cells, the filamentous phenotype remained (Fig. 4E). In contrast to our data showing that PBP3 is sufficient to promote division in Δ*mrcA* A. baumannii, PBP1A cannot rescue division when PBP3 is disrupted. Consistent with previous reports in E. coli (14, 39), PBP3 is an essential divisome component, where it regulates septal peptidoglycan biogenesis, and its activity cannot be compensated for by PBP1A in A. baumannii. Together, these data suggest that while both PBP1A and PBP3 interact in the divisome and possibly have some overlapping functions, they also have distinct roles in septal peptidoglycan biosynthesis. aPBP activity is hypothesized to competitively deplete lipid II precursors (1, 4, 5, 40). Therefore, PBP1A activity may more rapidly direct lipid II to sites of septal peptidoglycan biogenesis than FtsW/PBP3.

## DISCUSSION

Peptidoglycan biosynthesis is a validated target to treat Gram-negative infections, and recent studies have highlighted a key role for PBP1A in proper division and fitness in A. baumannii (23, 24, 41). These studies strongly imply that the canonical coccobacilli morphology associated with A. baumannii is largely dependent on PBP1A peptidoglycan synthase activity at the septum, an unexpected enzymatic role relative to well-studied Gram-negative model systems. In rod-shaped E. coli, PBP1A and PBP1B have distinct roles. PBP1A coordinates with elongasome to polymerize peptidoglycan along the side wall (14), and PBP1B interacts with the divisome to assemble septal peptidoglycan (42). However, disruption of either aPBP enzyme is compensated for by the other, indicating that they are functionally redundant (14). Despite the widely accepted dogma in Gram-negative bacteria, PBP1B activity cannot compensate for PBP1A in A. baumannii. Intriguingly, the PBP1A regulator LpoA, an outer membrane lipoprotein that stimulates synthase activity in E. coli, is not conserved in A. baumannii. Absence of the cognate regulator may support a unique role for PBP1A as a septal peptidoglycan synthase. In line with this hypothesis, manipulating PBP1A levels alone was sufficient to induce morphological changes in A. baumannii (Fig. 2). Further, PBP1A was enriched at the midcell during growth (Fig. 2), where we found that it directly interacts with PBP3, but we did not detect a direct PBP2-PBP1A interaction in the growth phase (Fig. 3). Together, these data support a model where PBP1A directly promotes septal peptidoglycan biosynthesis in A. baumannii.

We have also shown that PBP1A levels are dynamic, where high PBP1A levels in the stationary phase were evident relative to growth phase (Fig. 1D), suggesting that it may serve an additional role. Increased PBP1A levels may reflect a final septation event at the end of the growth phase before entry into the stationary phase. It is also possible that during the growth phase, PBP1A primarily interacts with the divisome to mediate septation, but increased levels in the stationary phase could also promote activity along the cell wall axis in complex with the elongasome or possibly as a repair complex (40, 43). In fact, previous work (44) suggested that PBP1A showed weak interactions with putative elongasome components (not including PBP2). However, there are two concerns with these data: the two-hybrid screen was not validated, and only direct interactions between PBP1A and PBP2 have been described in E. coli (17). Consistent with our previous analysis (23), another group also showed (41) that overexpression of PBP1A in wild-type cells induced cell rounding (Fig. 2C). While the other group speculated that PBP1A inhibits elongasome activity (41), it is also possible that PBP1A promotes an increased septation rate to promote the formation of short, round cells. It should also be acknowledged that overexpression of PBP1A is not necessarily an indication of increased activity by the outer membrane lipoprotein LpoA, as PBP1A traditionally requires an outer membrane lipoprotein activator in other Gram-negative bacteria (45). It is unknown how the elongasome and divisome complexes compete for lipid II precursors, but our studies here (Fig. 2A) suggest that recruitment of PBP1A to the midcell could shift the precursor pool toward septal peptidoglycan synthesis during growth, while indirectly inhibiting lipid II availability to the elongasome. Additional studies are warranted to better understand the outcomes of PBP1A activity on elongasome-dependent peptidoglycan biogenesis.

PBP1A overexpression was also not sufficient to compensate for aztreonam-inhibited PBP3 activity. Whereas PBP1A and PBP3 are both necessary for productive septation in wild-type A. baumannii, these findings indicate that the two septal peptidoglycan synthases have independent roles in division. Considering that PBP1A localizes to the site of septal peptidoglycan biogenesis prior to septum formation (Fig. 2B), it may support a model where PBP1A builds the recently described septal peptidoglycan wedge that forms after cellular constriction and prior to septation (4). Previous work suggested that septal peptidoglycan wedge formation prevents lysis during division (4) and may be one possible mechanism for the fitness defect we and others (24) have observed in Δ*mrcA* A. baumannii. Moreover, transpeptidase-inactivated PBP1A, but not PBP1B or PBP2, increased cellular lysis in A. baumannii cells under standard growth conditions (24), further supporting PBP1A as a major contributor to septal peptidoglycan biogenesis during division.

Another possible mechanism whereby PBP1A could contribute to divisome activity could be as an important component of the feedback network in peptidoglycan hydrolase activity. In E. coli, several elongasome, divisome, and hydrolytic enzymes, including PBP1A, are loosely associated with the outer membrane lipoprotein NlpI (46). NlpI is thought to act as a scaffold for both hydrolase and synthase complexes (46) and is important for regulating proteolysis of the hydrolytic endopeptidase MepS (47). NlpI phenotypes in E. coli have striking similarities to the A. baumannii PBP1A phenotype, where depletion leads to filamentation, and overexpression produces rounded, ovoid cells (48). While A. baumannii does not encode an NlpI homolog, the phenotypic similarities are intriguing and suggest that PBP1A may have an additional, indirect role in the localization or regulation of hydrolase activity. Consistent with this hypothesis, Δ*mrcA* cells form multiple septal sites, but septation is delayed, suggesting misregulation of hydrolase activity.

Not surprisingly, PBP3 (but not FtsW) overexpression rescued the division defect in Δ*mrcA* cells. PBP3 and FtsW are cooperative divisome partners; however, it is not completely unexpected that PBP3 overexpression alone would rescue the division defect. Monofunctional glycosyltransferases require transpeptidases for functionality (11), likely to prevent peptidoglycan polymerization without cross-linking if the transpeptidase is defective or absent. In this context, it is not surprising that overexpression of the transpeptidase PBP3 was capable of shifting peptidoglycan biogenesis toward division to phenotypically compensate for PBP1A inactivation, while overexpression of FtsW could not. While it is intriguing to speculate that PBP3 and FtsW work together to provide the major septal peptidoglycan polymerization activity, and PBP1A works to remodel the synthesized glycan chains, we previously demonstrated that the PBP1A glycosyltransferase activity is necessary for proper septation in A. baumannii (23), indicating a direct enzymatic role. Lastly, it is also possible that overexpression of FtsW did not increase the overall enzymatic activity. In Bacillus subtilis, YofA regulates FtsW (49); however, A. baumannii lacks a cognate YofA homolog.

PBP5 also rescued the Δ*mrcA* division defects in A. baumannii. PBP5 is a dd-carboxypeptidase found in both Gram-negative and Gram-positive bacteria that localizes to peptidoglycan biogenesis sites, where it forms tetrapeptides by cleaving the terminal d-alanine from its pentapeptide substrates (36, 50). While processing pentapeptides to tetrapeptides is important for peptidoglycan maturation, it is not clear why PBP5 overexpression resolves division defects in PBP3-depleted organisms (1, 36, 37), but these studies suggest that PBP5 could function similarly in A. baumannii. A recent report speculated that following PBP5-dependent tetrapeptide formation, an unidentified periplasmic ld-carboxypeptidase could modify periplasmic tetrapeptides to tripeptides (51), which were proposed to be the primary substrate of PBP3 (1, 52); however, increased PBP3 activity is dependent on lipid II availability, which presumably shifts peptidoglycan biogenesis toward division, away from elongation. If this pathway were intact in A. baumannii, it is possible that PBP5-dependent increases in tripeptide pools increase the substrate availability to PBP3 despite depletion, which could potentially rescue the division defect.

PBP1A expression prevents the selection of viable colistin-resistant lipooligosaccharide deficient (LOS-) A. baumannii (22). It was recently proposed that PBP1A may directly interfere with the elongasome activity by direct inhibition of PBP2 (41). Supporting this, our previous study (23) demonstrated that PBP1A overexpression promotes cell rounding. However, the more recent study (41) did not provide evidence for interactions between PBP1A and PBP2. In our studies, PBP2 levels in Δ*mrcA* cells were not higher than those in wild-type cells. However, a curious finding suggested that PBP2 overexpression in PBP1A-producing A. baumannii cells led to rapid lysis (data not shown). In contrast, PBP2 overexpression in Δ*mrcA* cells was tolerated (see Fig. S3C in the supplemental material). In Δ*mrcA* A. baumannii cells, PBP2 overexpression toxicity may be alleviated because competition from the divisome (lacking PBP1A) for lipid II substrate is reduced and septation is slowed. While these curious findings need to be explored further, preliminary data support a model where increased lipid II availability to the elongasome may result from defects in PBP1A activity. We plan to explore how this peptidoglycan regulatory mechanism promotes A. baumannii survival when the outer membrane is severely defective, because it could provide novel insights into antibiotic resistance mechanisms.

## MATERIALS AND METHODS

### Bacterial strains and growth.

All primers are listed in Table S1 in the supplemental material, and strains and plasmids are listed in Table S2. All A. baumannii strains were grown from freezer stocks initially on Luria-Bertani (LB) agar at 37°C. For selection, 25 μg/mL kanamycin was used when appropriate. Strains that harbored the pMMB plasmid for CoIP, complementation, or overexpression were supplemented with 25 μg/mL kanamycin and 2 mM isopropylthio-β-galactoside (IPTG).

### Fluorescent NADA staining.

As previously described (23, 27), overnight cultures were back diluted to an optical density at 600 nm (OD_600_) of 0.05 and grown at 37°C in Luria broth until they reached stationary or mid-logarithmic growth phase. The cells were washed once with Luria broth and normalized to an OD_600_ value of 1.0. An aliquot (3 μL) of 10 mM NBD-(linezolid-7-nitrobenz-2-oxa-1,3-diazol-4-yl)-amino-d-alanine (NADA) (Tocris Bioscience) was added to the resuspension. Cells were incubated with NADA at 37°C for 0.5 h. Following incubation, the cells were washed once and fixed with 1× phosphate-buffered saline (PBS) containing a (1:10) solution of 16% paraformaldehyde.

### Microscopy.

Fixed cells were immobilized on agarose pads and imaged using an inverted Nikon Eclipse Ti-2 widefield epifluorescence microscope equipped with a Photometrics Prime 95B camera and a Plan Apo 100× 1.45-numerical aperture objective lens, as previously described (23, 27). Green and red fluorescence images were taken using a filter cube with 470/40-nm or 560/40-nm excitation filters and 632/60-nm or 535/50-nm emission filters, respectively. Images were captured using NIS-Elements software.

### Image analysis.

All images were processed and pseudocolored using ImageJ Fiji (53), and the MicrobeJ plug-in was used for quantifications of pole-to-pole lengths (54). For pole-to-septa length quantifications, a segmentation code written for ImageJ was used. Cell length, width, and fluorescence data were plotted using Prism 9 (GraphPad 9.3.1). Demographs were generated using the MicrobeJ plugin. For the pole-to-septa lengths, 100 cells were analyzed; 450 cells were used for the demographs, and ≥300 cells were analyzed for all other experiments. Each experiment was independently replicated three times, and each replication was used in the data set. One representative image from the data sets was included in each figure.

### Growth curves.

Growth curves were calculated as previously described (23, 55, 56). Briefly, overnight cultures were back diluted to an OD_600_ value of 0.01 and set up as triplicate biological replicates in either 96- or 24-well plates (Brand; BrandTech). A BioTek Synergy Neo2 microplate reader was used to record the OD_600_ value, which was read every hour. The microplate reader was set to 37°C with continuous shaking. Growth curves were plotted using Prism 9. Each growth curve experiment was independently replicated three times, and one representative data set was reported.

### Western blotting.

Western blot analysis was carried out via gel transfer to polyvinylidene fluoride (PVDF) (Thermo Fisher Scientific). All blots were blocked in 5% milk for 2 h. The primary antibodies α-PBP1A, α-PBP3, α-PBP2, and α-RpoA were used at 1:1,000, 1:500, 1:300, and 1:1,000, respectively, followed by α-rabbit-horseradish peroxidase (HRP) secondary antibody at 1:10,000 (Thermo Fisher Scientific). SuperSignal West Pico Plus (Thermo Fisher Scientific) was used to measure the relative protein concentrations.

### Coimmunoprecipitation.

The protocol was adapted from previous work (33). Briefly, cultures were initially grown on LB agar overnight with incubation at 37°C. A single colony was used to inoculate 5 mL Luria broth, and the culture was grown overnight at 37°C. The overnight broth was diluted back to an OD_600_ value of 0.05 and grown to the mid-logarithmic growth phase at 37°C in 50 mL Luria broth. The cultures were collected, washed with 1× PBS, and incubated with 25 mM DSP (Thermo Fisher Scientific) in a total volume of 1 mL PBS for 1 h with shaking at 37°C. The reaction was quenched with room temperature incubation in 400 μL 1-M glycine with rocking for 15 min. The cells were lysed and solubilized overnight at 4°C with rocking. The lysate supernatant was centrifuged twice at 10,000 × *g* for 20 min, and the pellet was discarded. An aliquot (30 μL) of anti-FLAG M2 affinity resin (Sigma) was added to the supernatant and incubated overnight at 4°C with rocking. The resin was harvested at 6,000 × *g* and washed 4 times with radioimmunoprecipitation assay (RIPA) buffer. The pellet was resuspended in 100 μL 1× SDS-Page loading buffer with 5% β-mercaptoethanol (BME). The samples were boiled for 7 min and used for Western blotting.
