# Supplementary material for: PBP1A Directly Interacts with the Divisome Complex to Promote Septal Peptidoglycan Synthesis in Acinetobacter baumannii
Source: J Bacteriol. 2022 Nov 1;204(12):e00239-22. doi: 10.1128/jb.00239-22 (PMC9765026; doi:10.1128/jb.00239-22)
Supplement: Supplemental file 1 — Tables S1 and S2 and Fig. S1 and S3. Download jb.00239-22-s0001.pdf, PDF file, 0.8 MB [file jb.00239-22-s0001.pdf]

Supplemental material for 'PBP1A directly interacts with the divisome complex to promote septal peptidoglycan synthesis in *Acinetobacter baumannii*

Table S1: Primers used in this study

Primer	Sequence 5' -> 3'	Description	Used to make
RodA Forward	CGCGAGCTCATGTCTCCTAGTCCACA	[CGC]-[SacI]-[RodA]	pRodA
RodA Reverse	CGCGTCGACTTATCGATGTGTATGAATA	[CGC]-[SalI]-[RodA]	pRodA
FtsW Forward	CGCGGTACCATGGCAGGCTTA	[CGC]-[KpnI]-[Ftsw]	pFtsW
FtsW Reverse	CGCGTCGACTTAGAAGTTTGATTCTT	[CGC]-[Sal]-[Ftsw]	pFtsW
PBP2 Forward	CGCGGTACCATGAAACAGCACTTTCCT	[CGC]-[KpnI]-[PBP2]	$pPBP2_{OE}$
PBP2 Reverse	CGCGGATCCTTATTTATCATCATCATCTTTATAATCTTCATCGACCTCGTT	[CGC]-[BamHI]-[FLAG]-[PBP2]	$pPBP2_{OE}$
PBP3 Forward	CGCGGTACCATGGTAGATAAGCGAACAAAGCAAACACG	[CGC]-[KpnI]-[PBP3]	pPBP3-FLAG
PBP3 Reverse	CGCGTCGACTTACTTGTCGTCATCGTCTTTGTAGTCCCTGCGAATAGGAT	[CGC]-[SalI]-[FLAG]-[PBP3]	pPBP3-FLAG
PBP1A-FLAG Forward	CGCCTCGAGATGAAAAAGCTATCCAGTTTGGGCTT	[CGC]-[XhoI]-[PBP1A]	pPBP1A-FLAG
PBP1A-FLAG Reverse	CGCGGTACCTTATTTATCATCATCATCTTTATAATCCTCAATTTGATTAAT	[CGC]-[KpnI]-[FLAG]-[PBP1A]	pPBP1A-FLAG
pMMB sequencing Forward	CGGTTCTGGCAAATATTCTGAAA	Plasmid confirmation primer	pRodA, pFtsW, pPBP2-FLAG, pPBP3-FLAG, pPBP1A-FLAG
pMMB sequencing Reverse	GCCGCCAGGCAAATTCTGTT	Plasmid confirmation primer	RodA, pRodA, pFtsW, pPBP2-FLAG, pPBP3-FLAG, pPBP1A-FLAG
PBP2 FRT 5'	ACAAAGTCAAAAAAGCCAATTTACCTCTTCACTGAAACTTTGAAAAATATGCGATATTTTAAGTGCGCTTGCGTTAGAATAAGCAGCTATTTTTCTCAC	5' Recombineering primer	Δ*mrdA*

	CCTATGTCATTAAGTCGATCCGTATG		
PBP2 FRT 3’	CATGAGCCAGTTAAATAAAGTTTCGCGGACACGATCGGGAGTTGGTCTTAATCCTTCAATACTGGCGAATGGTAAAACTCGTCTTTTCCATTCGCCCCCAATAATGCGTAATTGATTTTTCATTA	3’ Recombineering primer	Δ*mrdA*
PBP2 Forward	GCTTACTGTCAAAACTGCAAGTAAGGG	Mutation confirmation primer	Δ*mrdA*
PBP2 Reverse	TGTTCTTTTAGGCGTGGTAAGGCT	Mutation confirmation primer	Δ*mrdA*

Table S2: Strains and plasmids used in this study

Strain/Plasmid	Description	Reference/Source
**Strains**		
A. baumannii ATCC 17978	wild type	ATCC (1)
A. baumannii ATCC 17978	Δ*mrcA*	(2)
A. baumannii ATCC 17978	Δ*mrcA*/pPBP1A	(2)
A. baumannii ATCC 17978	WT/pPBP1A$_{OE}$	(3)
A. baumannii ATCC 17978	Δ*mrcA*/pPBP1A$_{OE}$	(3)
A. baumannii ATCC 17978	Δ*mrcB*	(2)
A. baumannii ATCC 17978	Δ*mrcA*/pPBP1A-mCherry	(3)
A. baumannii ATCC 17978	Δ*mrcA*/pPBP1B$_{OE}$	This Study
A. baumannii ATCC 17978	Δ*mrcA*/pFtsW	This Study
A. baumannii ATCC 17978	Δ*mrcA*/pPBP2$_{OE}$	This Study
A. baumannii ATCC 17978	Δ*mrcA*/pPBP5	This Study
A. baumannii ATCC 17978	Δ*mrcA*/pPBP3$_{OE}$	This Study

A. baumannii ATCC 17978	WT/pPBP1A-FLAG	This Study
A. baumannii ATCC 17978	WT/pPBP3-FLAG	This Study
A. baumannii ATCC 17978	WT/pPBP3$_{OE}$	This Study
A. baumannii ATCC 17978	WT/pFtsW	This Study
A. baumannii ATCC 17978	WT/pPBP5	This Study
A. baumannii ATCC 17978	Δ*mrdA*	This Study
**Plasmids**		
pABBRKn	pABBR_ MCS with the Kn^R gene from pKD4 replacing the *bla* gene, Kn^R	(2)
pMMBKn	pMMBKn_MCS with the Kn^R gene from pKD4 replacing the *bla* gene, Kn^R	(2)
pABBRKn-mCherry	pABBRKn with the *mCherry gene* inserted into the KpnI and SacI sites, Kn^R	(3)
pPBP1A	pABBRKn with the *mrcA* gene and native promoter inserted into the XhoI and KpnI sites, Kn^R	(3)
pPBP1A$_{OE}$	pMMBKn with the *mrcA* gene inserted into the XhoI and KpnI sites, under an IPTG inducible promoter, Kn^R	(3)
pPBP1A-mCherry	pABBRKn-mCherry with the *mrcA* gene inserted into the XhoI and SacI sites, Kn^R	(3)
pAT03	pMMB67EH with FLP recombinase, Amp^R	(4)
pAT04	pMMB67EH with the REC$_{Ab}$ system, Tet^R	(4)
pKD4	Kn^R	(4)
pPBP1B$_{OE}$	pMMBKn with the *mrcB* gene inserted into the KpnI and SalI sites, Kn^R	(2)
pFtsW	pMMBKn with the *ftsW* gene inserted into the KpnI and SalI sites, Kn^R	This Study
pPBP5	pMMBKn with the *dacA* gene inserted into the KpnI and SalI sites, Kn^R	This Study
pPBP2$_{OE}$	pMMBKn with the *mrdA* gene with a FLAG tag inserted into the KpnI and BamHI sites, Kn^R	This Study
pPBP3$_{OE}$	pMMBKn with the *ftsI* gene with a FLAG tag inserted into the KpnI and BamHI sites, Kn^R	This Study

pPBP3-FLAG	pMMBKn with the *ftsI* gene with a FLAG tag inserted into the KpnI and SalI sites, Kn^R	This Study
pPBP1A-FLAG	pMMBKn with the *mrcA* gene with a FLAG tag inserted into the XhoI and KpnI sites, under an IPTG inducible promoter, Kn^R	This Study

Supplemental Figures

Figure S1

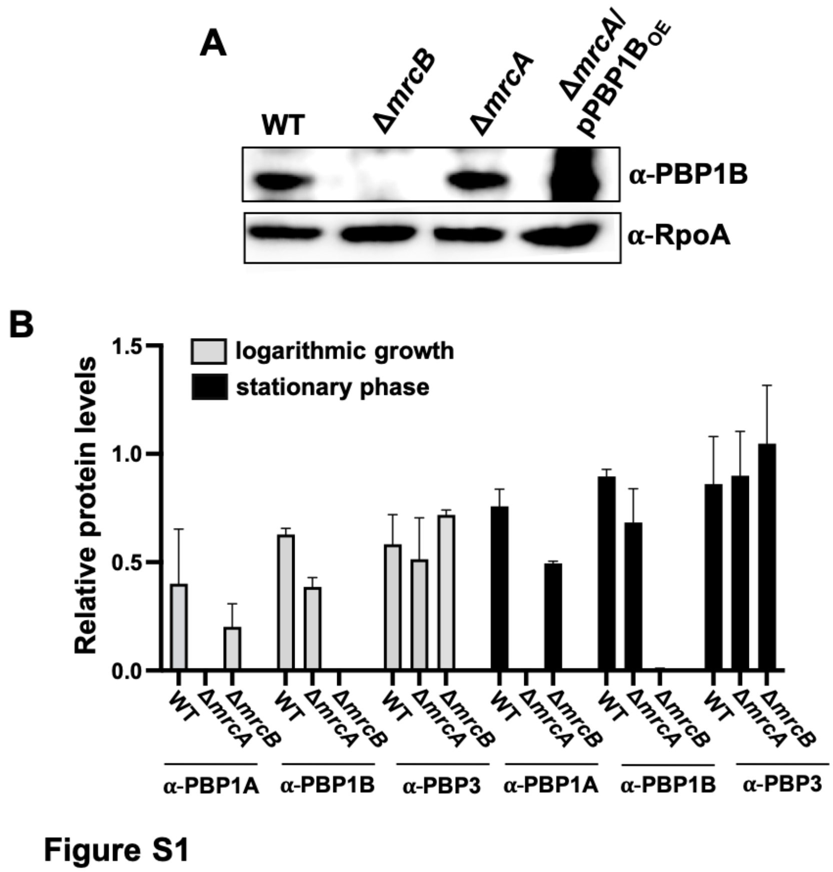

Figure S1: (A) PBP1B expression levels in *A. baumannii* wild type (WT) and mutants in growth phase. RpoA is the loading control. PBP1B is 88.21 kDa; RpoA is 37.62 kDa. (B) Densitometry of western blot analysis as calculated by ImageJ. Relative protein levels are represented as fold change over RpoA loading control. Error bars indicate the variance between two independent experiments. A representative image is shown in Fig 1D.

Figure S2

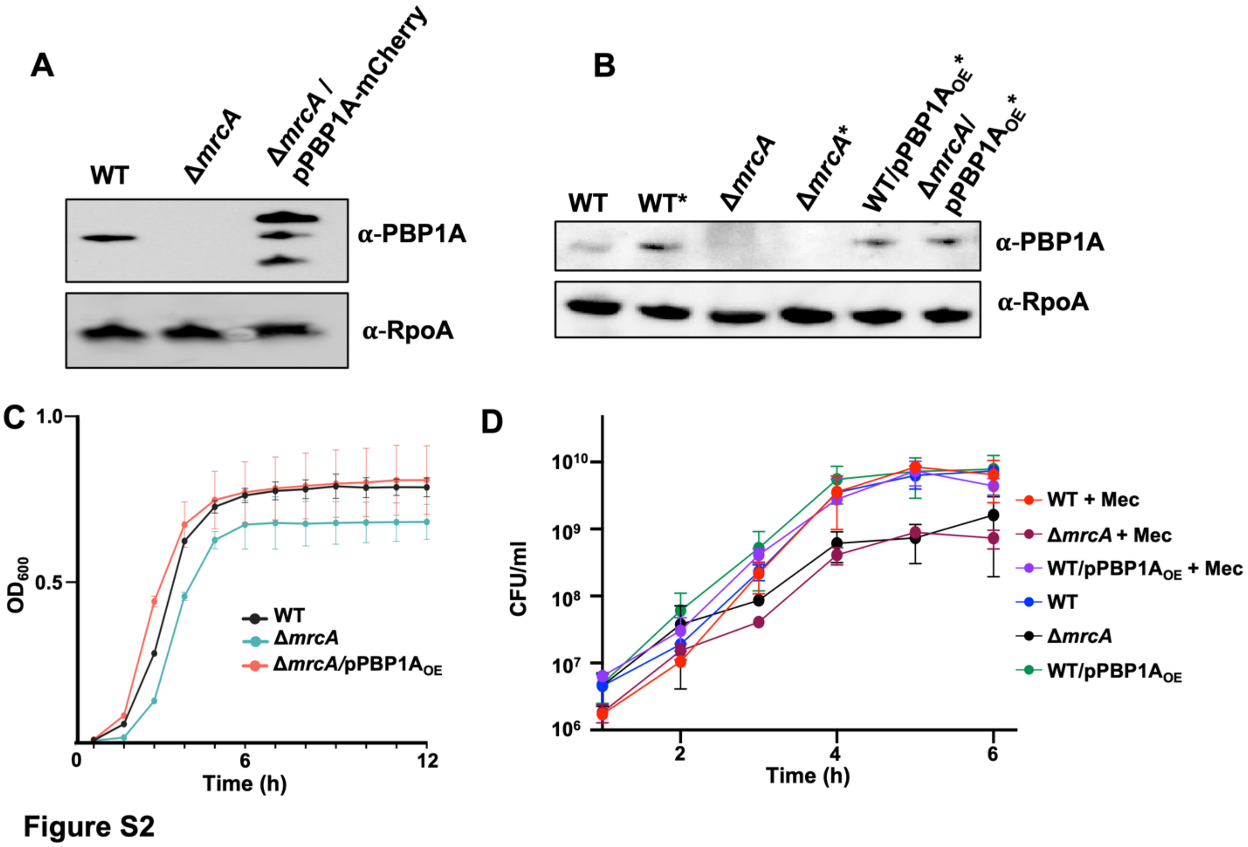

Figure S2: (A) PBP1A-mCherry expression under native promoter in wild type (WT) *A. baumannii.* The top band corresponds with the PBP1A-mCherry fusion predicted molecular weight. RpoA is the loading control. PBP1A is 94.74 kDa; PBP1A-mCherry is 120.01 kDa; RpoA is 37.62 kDa. (B) PBP1A expression in *A. baumannii* WT and mutants. * = treated with sub-MIC mecillinam (32 mg/L). RpoA is the loading control. (C) PBP1A overexpression restores the *mrcA* growth defect. Optical density growth curve of WT, Δ*mrcA,* and Δ*mrcA*/pPBP1A$_{OE}$ showing that PBP1A expression restores the growth (fitness) defect. Error bars represent standard deviation. (D) CFU/ml of WT, Δ*mrcA,* and WT/pPBP1A$_{OE}$ grown in the presence or absence of sub-MIC mecillinam (32 mg/L). Error bars represent standard deviation.

Figure S3

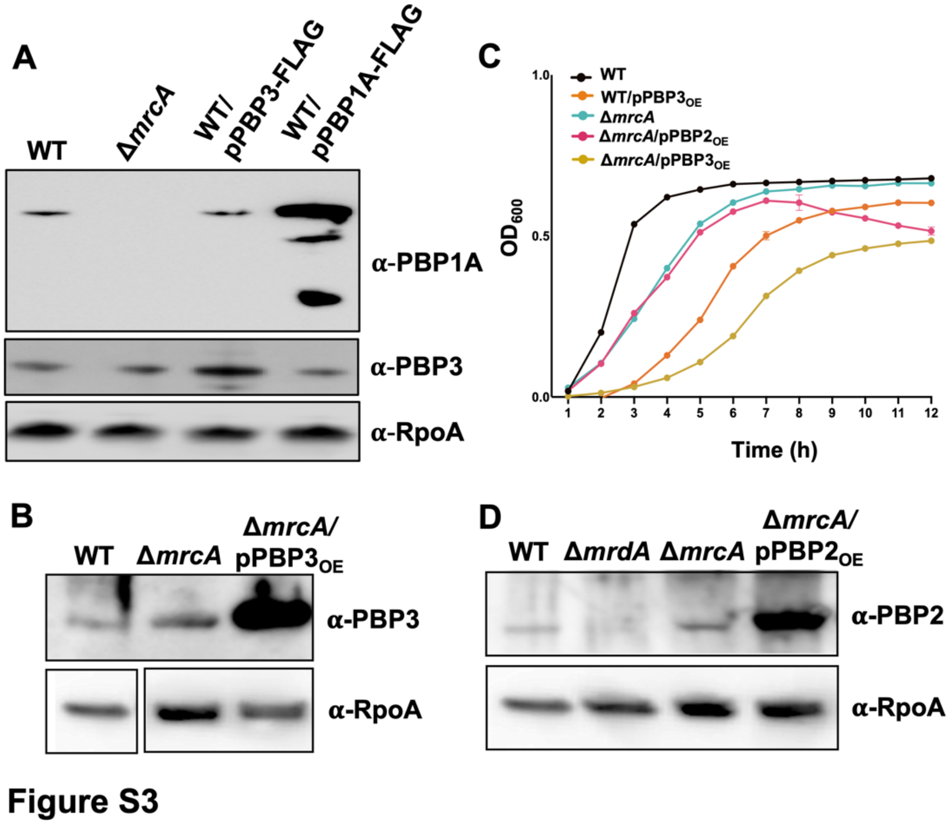

Figure S3: (A) Whole-cell lysates of FLAG fusion strains included in the co-immunoprecipitation to determine relative protein abundance. PBP1A is 94.74 kDa; PBP3 is 67.66 kDa; RpoA is 37.62 kDa. (B) PBP3 expression in *A. baumannii* wild type (WT) and mutants. (C) Growth curve of wild type and Δ*mrcA* overexpressing PBP3 and PBP2. Error bars represent standard deviation. (D) PBP2 expression in *A. baumannii* WT and mutants. PBP2 (encoded by *mrdA*) is 74.45 kDa.

References

1. Baumann P, Doudoroff M, Stanier RY. 1968. A study of the Moraxella group. II. Oxidative-negative species (genus *Acinetobacter*). *J Bacteriol* 95:1520–1541.

2. Boll JM, Crofts AA, Peters K, Cattoir V, Vollmer W, Davies BW, Trent MS. 2016. A penicillin-binding protein inhibits selection of colistin-resistant, lipooligosaccharide-deficient *Acinetobacter baumannii*. *Proc Natl Acad Sci USA* 113:E6228–E6237.

3. Kang KN, Kazi MI, Biboy J, Gray J, Bovermann H, Ausman J, Boutte CC, Vollmer W, Boll JM. 2021. Septal Class A Penicillin-Binding Protein Activity and LD -Transpeptidases Mediate Selection of Colistin-Resistant Lipooligosaccharide-Deficient *Acinetobacter baumannii*. *mBio* 1:e02185-20.

4. Tucker AT, Nowicki EM, Boll JM, Knauf GA, Burdis NC, Trent MS, Davies BW. 2014. Defining gene-phenotype relationships in *Acinetobacter baumannii* through one-step chromosomal gene inactivation. *mBio* 5:e01313-01314.
